# Supplementary material for: Body Weight, Physical Activity, and Risk of Cancer in Lynch Syndrome
Source: Cancers (Basel). 2021 Apr 13;13(8):1849. doi: 10.3390/cancers13081849 (PMC8069994; doi:10.3390/cancers13081849)
Supplement: Supplementary file 1 [file cancers-13-01849-s001.pdf]

cancers

MDPI

Supplementary Files

Body Weight, Physical Activity, and Risk of Cancer in Lynch Syndrome

Tero Sievänen, Timo Törmäkangas, Eija K. Laakkonen, Jukka-Pekka Mecklin, Kirsi Pylvänäinen, Toni T. Seppälä, Päivi Peltomäki, Sarianna Sipilä and Elina Sillanpää

Statistical Modeling of Cancer Incidence

The exposures of interest consisted of weight as a continuous measure and physical activity as a binary measure. Both weight and physical activity were recollected backwards from date of cancer occurrence in ten-year intervals, so that for a subject the exposure data is structured in measurements starting from age 20 and continuing in 10-year intervals up to occurrence of cancer at T_i as shown in Figure S1 (panel A). The longitudinal trajectories for physical activity and weight are shown in Figures 3 and 4, respectively. For the responses to supervised physical activity question we used a statistical technique called jittering to avoid overplotting the category trajectories.

We used the counting process data format to model the recalled levels of weight and physical activity as time-dependent variables in the relative risk model (see e.g., Therneau and Grambsch 2000) adapted for random effects. Family structure was controlled for using a random effect structure with the coxme-function (library coxme, version 2.2-14) in R programming environment. The hazard function of the model can be expressed as:

$$\lambda(t_{i,j}) = \lambda_0(t)\exp(\mathbf{x}_{ij}^{\mathrm{T}}\boldsymbol{\beta} + u_j + v_{ij});\ i = 1, \ldots, n_j,\ j = 1, \ldots, N$$

where N is the number of families, n_j the number of subjects in family j, t indexes the unit of time, $\lambda_0(t)$ is the baseline hazard function, $\mathbf{x}_{ij}$ is a p x 1 vector of fixed covariates for individual i in family j, and $\boldsymbol{\beta}$ is the corresponding p-dimensional vector of regression coefficients, u_j is a family-specific random intercept accounting for familial excess risk among subjects given family and v_{ij} is random effect for subject i within family j accounting for excess risk among subject variability within family. To determine identifiability of the model parameters, we assumed that the random effects followed the log-normal distribution with zero-means and variances estimated from the data. Parameter estimation was based on the method of partial maximum likelihood penalized for the random effects.

The impact of the main predictors on cancer risk were assessed in separate models for types of cancer and separately for men and women. We also used separate models for the main exposures weight and physical activity. The values of these variables were permitted to vary in ten-year intervals according to the weight and physical activity patterns as they were recalled by the subjects. The crude model effects of the linear predictor included the fixed effects:

$$\mathbf{x}_i{}^{\mathrm{T}}\boldsymbol{\beta} = \beta_X X_{i(t)}, \quad (1)$$

where $X_{i(t)}$ is the value observed for weight or physical activity, respectively. We assumed that the regression coefficient β_X did not vary over time, i.e., we assumed that proportionality of hazards holds for cancer incidence with respect to the predictors of interest. To assess the tenability of this assumption we used plots and test based on scaled Schoenfeld residuals (see Grambsch and Therneau 1994) and found no substantial evidence of change in the ratio of cancer incidence with respect to the covariates of interest (see Table S3). For the adjusted model we added the effects from additional fixed covariates in the linear predictor. Model covariates are described in more detail in the main article.

Exposure data included missing data. If data was missing in the middle or at the end of the follow-up period, we used the preceding values as best guesses of the missing exposure data. If data was missing from the beginning of the follow-up period, we replaced these observations with the earliest available data. If all exposure data was missing, cases were excluded from analysis. Missing data treatment and patterns of missing data are shown in Figure S2 and Table S2.

Although we found no evidence suggesting non-proportionality of hazards (see Tables S3 and S4), we performed additionally analyses to assessed whether there might be risk modification from the last recalled weight and physical activity measurement prior to diagnosis of cancer. For this analysis we dropped the data from the preceding measurements and used only the last timepoint measurements (see Figure S1, panel B). In this case time can be considered in two ways, either by using the counting process form as the interval $[t_L, t_F)$, where t_L is the last measurement before end of follow-up and t_F is the time for end of follow-up (cancer diagnosis or censoring) or as the length of that interval as $[0, t\text{-}t_L)$. The formulation of time intervals for analysis is the only distinguishing feature between this and the previous model. It should also be noted that in this analysis weight and physical activity are no longer time-dependent covariates because we only utilize measurements from one interval. Also, in this model one should account for the fact that subjects are of different age when cancer occurs. This occurs automatically in the counting process formulation, but at least three alternatives can be considered for the length-of-interval approach: 1) consider no impact from age, 2) use age for the start of interval as a predictor effect, and 3) stratify by age. For our current analysis, all three methods yield similar results. Finally, it should be noted that this analysis does not provide a means to assess the significance of differences in predictor effects with respect to analyses based on model (1), but instead these comparisons should be considered tentative. The coefficients from all models are shown in Table S5 (age-stratified results are omitted because these results are equivalent to the counting process formulated model). Table S1 lists the specifications for the models of these additional analyses.

Table S1. Model specification details.

Time	Model	Age-Adjustment
$[t_L, t_F)$	1	Included in expression of time
$[0, t\text{-}t_L)$	2	None
$[0, t\text{-}t_L)$	3	Start of period, t_L, as predictor
$[0, t\text{-}t_L)$	Equivalent to 1	Start of period, t_L, forms strata

Model number is used as reference in supplementary tables 2 and 3. In supplementary table 2 we omit results of proportionality of hazards tests for models 2 and 3 due to similarity to model 1.

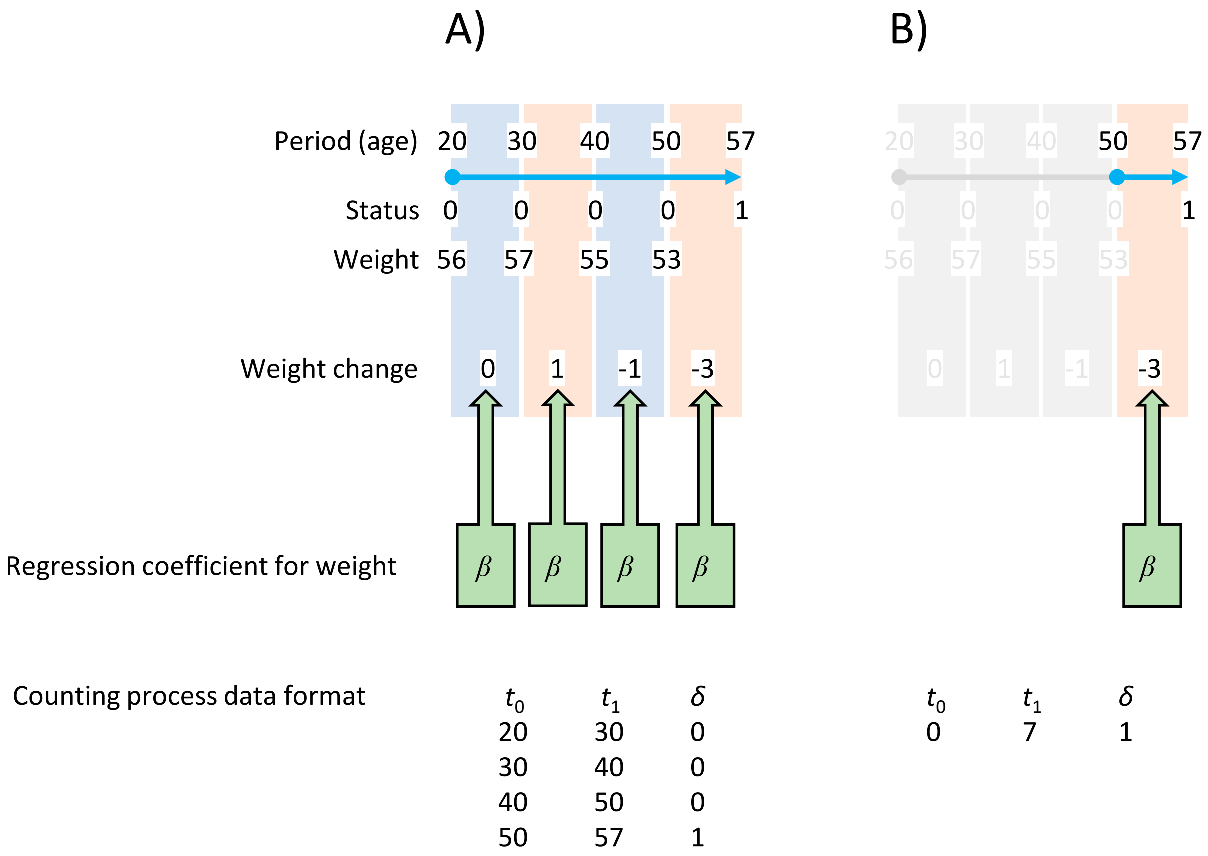

Figure S1. Modelling time-dependent predictors and effects. For the example patient cancer was observed at age 57. Weight was recollected in full ten-year intervals starting from age 20. As we found no evidence of non-proportionality, hazards were considered proportional and hence the regression coefficient retains the same value over the complete follow-up period. Panel (**A**) shows data considered for full longitudinal analysis and (**B**) data for analysis focusing only on the last time interval prior to study end. The data format required for the counting process analysis is shown below both analysis settings.

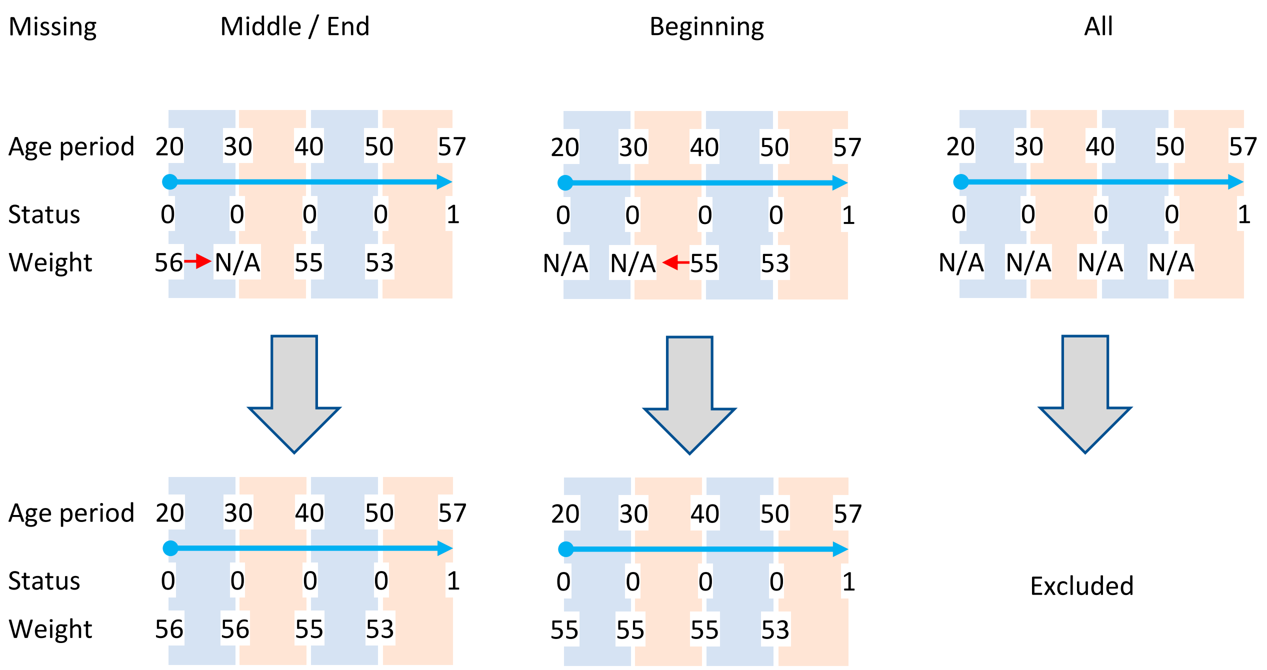

Figure S2. Treatment and observed of missing data patterns. Three types of missing data patterns are illustrated for patients whose cancer was observed at age 57.

Table S2. Missing data patterns.

Missing Pattern	Weight				Physical Activity			
	Men		Women		Men		Women	
	N	(%)	*N*	(%)	*N*	(%)	*N*	(%)
None	135	(76.9)	158	(70.9)	137	(77.8)	184	(82.5)
Beginning	6	(3.4)	6	(2.7)	27	(15.3)	15	(6.7)
Middle/end	9	(5.1)	32	(14.3)	2	(1.1)	12	(5.4)
All	26	(14.8)	27	(12.1)	10	(5.7)	12	(5.4)

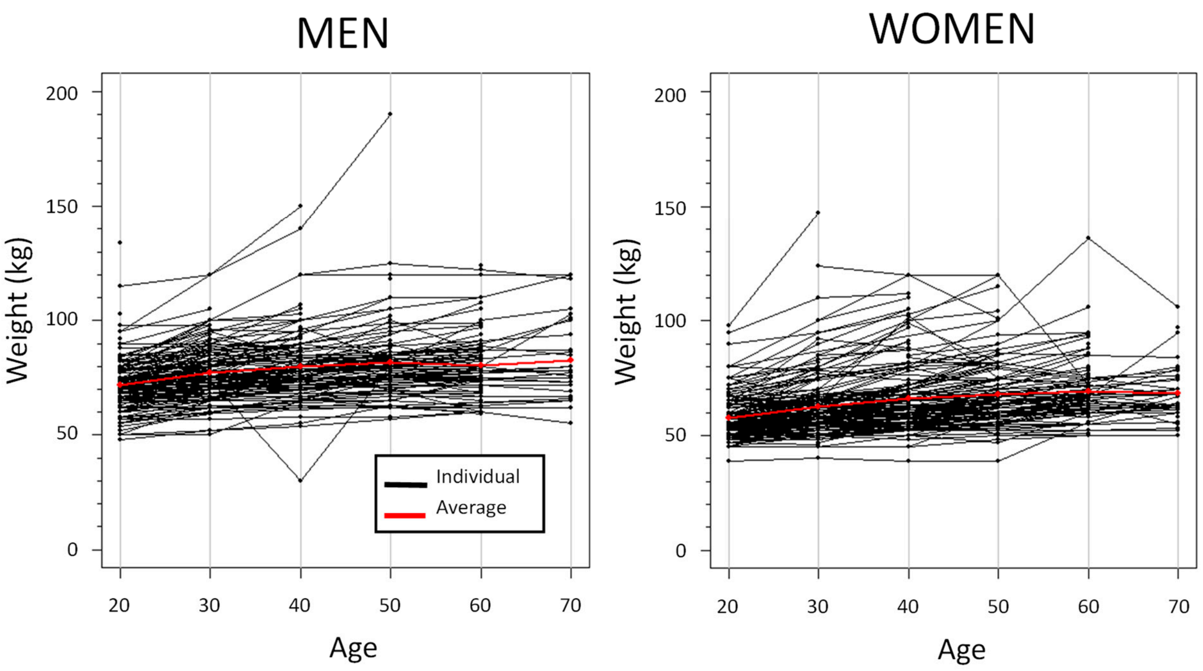

Figure S3. Individual and average recalled weight trajectories for men and women.

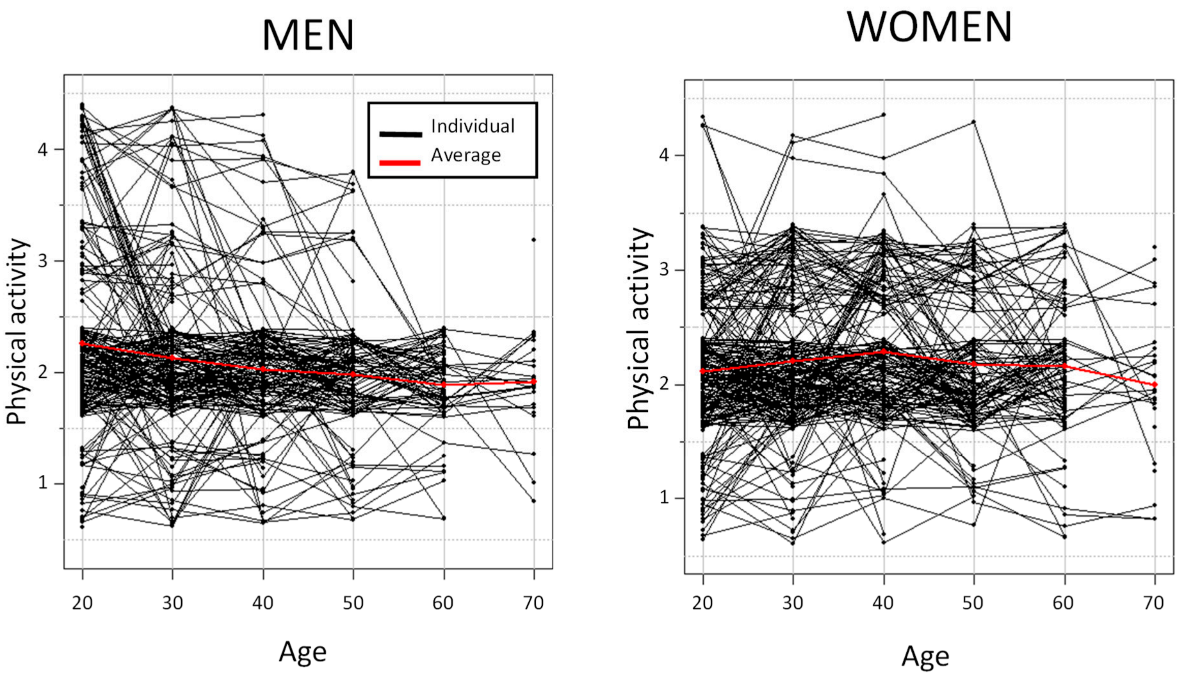

Figure S4. Individual and average trajectories recalled participation in organised physical activity based on the original Figure 0. 4] was added to each activity value). Response categories: 4 Regular training for competitive sports; 3 Regular other supervised exercise in a sports club or similar; 2 Regular independent leisure time physical activity; 1 I have not participated in physical activity.

Table S3. Tests of proportionality of hazards based on scaled Schoenfeld residuals for models utilizing longitudinal trajectories of weight and physical activity as time-dependent predictors.

**Predictor variable**	**Model**	**Crude**			**Adjusted**		
		χ^2	**df**	***p*-Value**	χ^2	**df**	***p*-Value**
Weight	Men/All cancers	0.83	3	0.841	1.42	20	1.000
	Men/CR-cancer	3.57	3	0.312	4.10	20	1.000
	Women/All cancers	0.13	3	0.988	0.59	19	1.000
	Women/CR-cancer	0.57	3	0.904	1.45	19	1.000
PA	Men/All cancers	0.01	3	1.000	7.7×10^{-5}	19	1.000
	Men/CR-cancer	10^{-3}	3	1.000	0.20	19	1.000
	Women/All cancers	0.40	3	0.942	0.95	18	1.000
	Women/CR-cancer	1.05	3	0.790	1.71	18	1.000

Note. χ^2: test statistic for proportionality of hazards; *df*: degrees of freedom.

Table S4. Tests of proportionality of hazards based on scaled Schoenfeld residuals for models utilizing last measurement interval weight and physical activity as predictors.

Predictor variable	Model	Crude			Adjusted		
		χ^2	*df*	*p*-Value	χ^2	*df*	*p*-Value
	Model 1						
Weight	Men/All cancers	1.31	1	0.252	1.72	20	1.000
	Men/CR-cancer	3.12	1	0.077	3.39	20	1.000
	Women/All cancers	0.83	1	0.361	0.94	19	1.000
	Women/CR-cancer	0.13	1	0.718	0.19	19	1.000
PA	Men/All cancers	1.06	3	0.787	3.61	19	1.000
	Men/CR-cancer	0.66	3	0.883	2.69	19	1.000
	Women/All cancers	3.23	3	0.357	6.39	18	0.994
	Women/CR-cancer	2.59	3	0.460	4.42	18	1.000
	Model 2						
Weight	Men/All cancers	1.31	1	0.250	1.72	20	1.000
	Men/CR-cancer	3.12	1	0.077	3.39	20	1.000
	Women/All cancers	0.83	1	0.360	0.94	19	1.000
	Women/CR-cancer	0.13	1	0.720	0.19	19	1.000
PA	Men/All cancers	1.06	3	0.790	3.61	19	1.000
	Men/CR-cancer	0.66	3	0.880	2.69	19	1.000
	Women/All cancers	3.23	3	0.357	6.39	18	0.994
	Women/CR-cancer	2.59	3	0.460	4.42	18	1.000

Note. χ^2: test statistic for proportionality of hazards; *df*: degrees of freedom.

Table S5. Summary of hazard ratios (HR) and 95 % confidence intervals from various models.

Predictor variable	Model	Crude			Adjusted		
		HR	Lower	Upper	HR	Lower	Upper
Weight	Men/All cancers	1.01	1.00	1.03	1.02	1.00	1.04
	1	0.99	0.98	1.01	0.99	0.97	1.01
	2	1.00	0.98	1.01	1.00	0.98	1.02
	3	1.00	0.98	1.01	1.00	0.98	1.02
	Men/CR-cancer	1.02	1.00	1.03	1.03	1.01	1.05
	1	1.00	0.98	1.01	1.00	0.98	1.02
	2	1.00	0.98	1.02	1.01	0.99	1.03
	3	1.00	0.98	1.02	1.01	0.99	1.03
	Women/All cancers	0.99	0.97	1.01	1.00	0.98	1.02
	1	1.01	1.00	1.03	0.98	0.96	1.00
	2	0.98	0.97	1.00	0.98	0.96	1.00
	3	0.98	0.97	1.00	0.98	0.96	1.00
	Women/CR-cancer	0.99	0.96	1.01	0.99	0.96	1.02
	1	0.98	0.96	1.00	0.95	0.92	0.99
	2	0.98	0.95	1.00	0.96	0.93	1.00
	3	0.98	0.95	1.00	0.97	0.93	1.00
PA	Men/All cancers	0.44	0.19	1.04	0.38	0.15	0.98
	1	0.69	0.29	1.64	0.74	0.27	2.01
	2	0.78	0.34	1.80	0.92	0.36	2.38
	3	0.79	0.34	1.81	0.90	0.35	2.32
	Men/CR-cancer	0.57	0.25	1.33	0.52	0.20	1.36
	1	0.93	0.39	2.24	0.99	0.36	2.73
	2	1.00	0.43	2.32	1.07	0.41	2.78
	3	0.99	0.42	2.29	1.02	0.39	2.68
	Women/All cancers	1.31	0.86	1.97	1.26	0.79	2.01
	1	1.16	0.76	1.76	1.15	0.70	1.90
	2	1.36	0.90	2.05	1.44	0.89	2.32
	3	1.31	0.86	1.99	1.40	0.87	2.24
	Women/CR-cancer	1.16	0.65	2.10	1.28	0.65	2.52
	1	0.92	0.49	1.72	0.99	0.48	2.02
	2	1.10	0.60	2.03	1.33	0.67	2.61
	3	1.08	0.59	2.00	1.29	0.66	2.53

Model 1: risk during last observation time, 2: time as length of last measurement interval, no age adjustment, 3: time as length of last measurement interval time adjustment.

Table S6. Summary of the participants' physical activity categories throughout the retrospective follow-up.

Physical Activity within Age Category	Men	Women
20–29 years	*N* = 202	*N* = 224
No regular physical activity [n (%)]	24 (11.9)	31 (13.8)
Regular independent leisure-time physical activity [n (%)]	128 (63.4)	139 (62.1)
Regular goal-oriented competitive sport [n (%)]	26 (12.9)	3 (1.3)
Other regular supervised physical activity [n (%)]	24 (11.9)	51 (22.8)
30–39 years	*N* = 189	*N* = 222
No regular physical activity [n (%)]	25 (13.2)	18 (8.1)
Regular independent leisure-time physical activity [n (%)]	128 (67.7)	143 (64.4)
Regular goal-oriented competitive sport [n (%)]	14 (7.4)	3 (1.4)
Other regular supervised physical activity [n (%)]	22 (11.6)	58 (26.1)
40–49 years	*N* = 165	*N* = 189
No regular physical activity [n (%)]	20 (12.1)	11 (5.8)
Regular independent leisure-time physical activity [n (%)]	127 (77.0)	117 (61.9)
Regular goal-oriented competitive sport [n (%)]	7 (4.2)	4 (2.1)
Other regular supervised physical activity [n (%)]	11 (6.7)	57 (30.2)
50–59 years	*N* = 131	*N* = 156
No regular physical activity [n (%)]	17 (13.0)	11 (7.1)
Regular independent leisure-time physical activity [n (%)]	104 (79.4)	107 (68.6)
Regular goal-oriented competitive sport [n (%)]	5 (3.8)	1 (0.6)
Other regular supervised physical activity [n (%)]	5 (3.8)	37 (23.7)
60–69 years	*N* = 81	*N* = 104
No regular physical activity [n (%)]	9 (11.1)	9 (8.7)
Regular independent leisure-time physical activity [n (%)]	72 (88.9)	69 (66.3)
Regular goal-oriented competitive sport [n (%)]	-	-
Other regular supervised physical activity [n (%)]	-	26 (25.0)
70+ years	*N* = 23	*N* = 24
No regular physical activity [n (%)]	3 (13.0)	5 (20.8)
Regular independent leisure-time physical activity [n (%)]	19 (82.6)	14 (58.3)
Regular goal-oriented competitive sport [n (%)]	-	-
Other regular supervised physical activity [n (%)]	1 (4.3)	5 (20.8)

Missing (n), 20-29 years: 39; 30-39 years: 54; 40-49 years: 111; 50-59 years: 178; 60-69 years: 280; 70+ years: 408.

References

1. Laird, A.K.; Dynamics of Tumour Growth, *Br J Cancer* **1964**, *13*, 490–502.
2. Grambsch, P.M., Therneau, T.M.; Proportional hazards tests and diagnostics based on weighted residuals. *Biometrika* **1994**, *81*, 515–526.
3. Therneau, T. M., Grambsch P. M.; Modeling Survival Data: Extending the Cox Model. *Springer*: New York.
4. Therneau, T.M.; coxme: Mixed Effects Cox Models. R package version 2.2-14. https://cran.r-project.org/package=coxme. Initially accessed on August, 2, 2019.
